# MicroRNA-93 Regulates Hypoxia-Induced Autophagy by Targeting ULK1

**DOI:** 10.1155/2017/2709053

**Published:** 2017-10-03

**Authors:** Wen Li, Yue Yang, Zhaoyu Ba, Shupeng Li, Hao Chen, Xiaoyan Hou, Linlin Ma, Pengcheng He, Lei Jiang, Longxuan Li, Rongrong He, Liangqing Zhang, Du Feng

**Affiliations:** ^1^Key Laboratory of Protein Modification and Degradation, School of Basic Medical Sciences, Affiliated Cancer Hospital & Institute of Guangzhou Medical University, Guangzhou Medical University, Guangzhou 511436, China; ^2^Anti-Stress and Health Research Center, College of Pharmacy, Jinan University, Guangzhou 510632, China; ^3^Department of Anesthesiology, Guangdong Medical University, Zhanjiang 524001, China; ^4^Department of Spine Surgery, Shanghai East Hospital, Tongji University School of Medicine, Tongji University, Shanghai 200120, China; ^5^Institute of Neurology, Guangdong Key Laboratory of Age-Related Cardiac-Cerebral Vascular Disease, Affiliated Hospital of Guangdong Medical University, Zhanjiang, Guangdong 524001, China; ^6^Department of Obstetrics and Gynecology, Beijing Hospital, National Center of Gerontology, Beijing 100730, China; ^7^Department of Cardiology, Guangdong Cardiovascular Institute, Guangdong Provincial Key Laboratory of Coronary Heart Disease Prevention, Guangdong General Hospital, Guangdong Academy of Medical Sciences, Guangzhou, China; ^8^Department of Neurology, Gongli Hospital, Pudong New Area, Shanghai 200135, China

## Abstract

The expression of the core autophagy kinase, Unc51-like kinase 1 (ULK1), is regulated transcriptionally and translationally by starvation-induced autophagy. However, how ULK1 is regulated during hypoxia is not well understood. Previously, we showed that ULK1 expression is induced by hypoxia stress. Here, we report a new ULK1-modulating microRNA, miR-93; its transcription is negatively correlated with the translation of ULK1 under hypoxic condition. miR-93 targets ULK1 and reduces its protein levels under hypoxia condition. miR-93 also inhibits hypoxia-induced autophagy by preventing LC3-I to LC3-II transition and P62 degradation; these processes are reversed by the overexpression of an endogenous miR-93 inhibitor. Re-expression of ULK1 without miR-93 response elements restores the hypoxia-induced autophagy which is inhibited by miR-93. Finally, we detected the effects of miR-93 on cell viability and apoptosis in noncancer cell lines and cancer cells. We found that miR-93 sustains the viability of MEFs (mouse embryonic fibroblasts) and inhibits its apoptosis under hypoxia. Thus, we conclude that miR-93 is involved in hypoxia-induced autophagy by regulating ULK1. Our results provide a new angle to understand the complicated regulation of the key autophagy kinase ULK1 during different stress conditions.

## 1. Introduction

Autophagy is a regulated and highly conserved process that can develop double-membraned autophagosomes to degrade large protein aggregates and damaged organelles upon fusing with lysosomes [1, 2]. Autophagy can not only recycle intracellular energy resources when the cell is deficient in nutrients but also remove cytotoxic proteins and damaged organelles under a lot of stress conditions including pathogen invasion, hypoxia, and mitochondrial depolarization [3–5].

ULK1 is a mammalian homologue of yeast Atg1 [6, 7]. As an initiator of autophagy, ULK1 regulates autophagy through interaction of upstream mTOR and AMPK and then transduce signals to downstream mediators [8–13]. ULK1 protein level can be regulated by a variety of stimuli such as nutrient-depleted condition, energy deprivation, and hypoxia [14–16]. In the initial stage of nutrient-depletion condition, the elevated transcription of ULK1 is immediately induced, but after prolonged starvation, the ULK1 protein level is reduced because of the suppressed translation as well as the increased degradation of ULK1, which limits further autophagy induction [17–19]. ULK1 level is significantly increased in response to inhibition of mitochondrial respiratory complexes in cell treated with inhibitors of the mitochondrial complex I (rotenone), complex II (thenoyltrifluoroacetone/TTFA), or complex III (antimycin A or myxothiazol) [17]. Previously, we also found that ULK1 level is induced in cells subjected to 1% oxygen stress condition or by mitochondrial depolarized drug, FCCP, indicating that the ULK1 level is regulated at both transcriptional and translational levels and is more complicatedly controlled than previously thought [15, 16].

miRNAs are 17 ~ 22 nucleotide, noncoding and single-stranded RNA molecules playing a role in a variety of pathophysiologic processes including apoptosis, cell proliferation, and differentiation [20–22]. It targets multiple genes via blocking mRNA translation. Silencing Dicer1, a major component of the miRNA-silencing machinery, increases the levels of the Atg protein and the formation of autophagosomes in cells [23]. Kap1, also known as TRIM28 (tripartite motif protein 28), acts as a scaffold for a multimolecular complex that silences transcription through the formation of heterochromatin [24, 25]. Hematopoietic-restricted deletion of Kap1 fails to induce mitophagy (the process of removing damaged mitochondrial through autophagy)-associated genes [26, 27] and retains mitochondria due to lack of repression of a subset of microRNAs, which target mitophagy transcripts. We also found that hypoxia-responsive miR-137 regulates mitophagy by targeting two mitophagy receptors FUNDC1 and NIX. These findings indicate that miRNAs can regulate autophagy [28, 29]. Although several studies on microRNA regulation of ULK1 have been reported, those microRNAs do not respond to hypoxic environments [30–38].

miR-93 plays a variety of roles in regulating cellular homeostasis. For example, the expression of miR-93 causes hypersusceptibility to vesicular stomatitis virus infection in Dicer1-deficient mice [39]. miR-93 promotes the axon growth of spinal cord neurons by targeting to EphA4 [40]. In proliferation, miR-93 promotes the proliferation of osteosarcoma cells by targeting PTEN [41, 42].

Nevertheless, how microRNA-modulated key autophagic machinery is involved in hypoxic condition remains poorly understood. Here, we uncovered the regulating mechanism between miRNA-93, ULK1, and hypoxia-induced autophagy.

## 2. Materials and Methods

### 2.1. Reagents and Antibodies

DMEM (Gibco, C11965500BT), fetal bovine serum (Gibco, 16000044), EBSS (MACGENE, CC026), FITC Annexin V apoptosis detection kit I (BD Biosciences, 556547), cell counting kit (YEASEN, QF0026), lysis buffer (Beyotime, P0013), PVDF membranes (Millipore, ISEQ00010), albumin bovine V (Solarbio, A8020), lipofectamine 2000 (Invitrogen, 11668027), Dual-Luciferase^®^ Reporter Assay System (Promega, E1910), and terminal deoxynucleotidyl transferase-mediated dUTP nick-end labeling (TUNEL) staining kit (Promega, G3250). Bafilomycin A1 was purchased from Sigma. The following antibodies were used: anti-ULK1 (Sigma-Aldrich, A7481), anti-P62 (MBL, PM045), anti-P62 (Abcam, ab56416), anti-LC3B (Sigma, L7543), anti-c-Myc monoclonal antibody (Sigma, C3956), anti-*β*-actin (Sino Biological, 100162-RP02-100), anti-Gapdh (Transgen, HC301), anti-ULK1 (Sigma, A7481), anti-ATG5 (Sigma, A0731), anti-ATG7 (Sigma, A2856), anti-Beclin1 (CST, 3738), anti-Tom20 (BD Biosciences, 612278), DAPI (CST, 4083), HRP Affinipure goat anti-mouse IgG (Earthox, E030110), and HRP Affinipure goat anti-rabbit IgG (Earthox, E030120). The following fluorescent secondary antibodies were used: Alexa Fluor 555-labeled donkey anti-mouse IgG antibodies (Invitrogen, A31570) and Alexa Fluor 488-labeled donkey anti-rabbit IgG antibodies (Invitrogen, A21206).

### 2.2. Cell Culture, Hypoxia, Starvation Treatment, and Transfection

The methods we used in the Materials and Methods were followed from our previous paper by Li et al. [43]. MEFs (mouse embryonic fibroblasts), CHO (Chinese hamster ovary cell), and HeLa cells were used in this study. Cells were cultured in Dulbecco's modified Eagle's medium supplemented with 10% FBS and 1% penicillin/streptomycin (Beyotime, referred to as complete medium) at 37°C and 5% CO_2_. To determine the levels of autophagy, we starved cells for different times with EBSS. Hypoxic conditions were achieved with a hypoxia chamber (Billups-Rothenberg) flushed with a preanalyzed gas mixture of 1% O_2_, 5% CO_2_, and 94% N_2_. Cell transfection was performed using lipofectamine 2000 according to protocols provided by manufacturers.

### 2.3. Plasmids

Myc-ULK1 constructs were described previously. The 3′ untranslated region (UTR) of ULK1 (NM_009469) was amplified from mouse cDNA. The primers used were as follows: 5′-GCGCTAGCCCAGGGGTCCCTTGCCCAC-3′ (UTR, F), 5′-GGTCTAGAGTAAAGTGTGGAAGTTGAGG-3′ (UTR, R). PCR products were cloned into pmirGLO dual-luciferase miRNA target expression vector (Promega) using NheI and XbaI restriction sites, named pmirGLO-ULK1. The corresponding binding site mutation was also cloned using the following primers: 5′-TTTGTAAGTCACCGGTAACTGCCATGCATACAGAGACTGGA-3′ (M-UTR, F), 5′-TCCAGTCTCTGTATGCATGGCAGTTACCGGTGACTTACAAA-3′ (M-UTR, R). Mutation, named mutant pmirGLO-ULK1, was created by site-directed mutagenesis, changing “gcacttt” to “aactgcc.” Q5^®^ High-Fidelity DNA Polymerase (Bio Labs, M0491S) was used for PCR reaction. The plasmids were sequenced to verify the accuracy.

### 2.4. siRNAs and miRNAs

Five pairs of siRNAs were designed and synthesized as follows by GenePharma: si-ULK1-Mus-488 (sense: CCGUCAAAUGCAUUAACAATT, antisense: UUGUUAAUGCAUUUGACGGTT), si-ULK1-Mus-2457 (sense: GCUGCUUAAGGCUGCAUUUTT, antisense: AAAUGCAGCCUUAAGCAGCTT), si-ULK1-Mus-3310 (sense: GCUGUGCAAAUGGUACAAUTT, antisense: AUUGUACCAUUUGCACAGCTT), and negative control (NC) and negative control FAM (sense: UUCUCCGAACGUGUCACGUTT, antisense: ACGUGACACGUUCGGAGAATT). miR-93 mimics (93) and miR-93 inhibitor (IN-93) were synthesized by RiboBio (Guangzhou, China) with reference to miRNA's sequence information in miRbase. Meanwhile, NControl (NC) and inhibitor NControl (IN-NC) were also synthesized as a negative control.

### 2.5. Luciferase Assay

Cells were seeded in 24-well plates 1 day before transfection. For reporter assays, the cells were transiently cotransfected with 0.8 *μ*g of reporter plasmid in the presence of 100 nM scramble NC (NC), miR-93 mimics (93), NC inhibitor (IN-NC), or miR-93 inhibitor (IN-93) using lipofectamine 2000. Firefly and Renilla luciferase activities were measured consecutively by using dual-luciferase reporter assay system according to the manufacturer's instructions. Independent experiments were performed in triplicate.

### 2.6. qRT-PCR

Total RNA was isolated with TRIzol (Life Technologies) followed by a DNase treatment to eliminate contaminating genomic DNA (Thermo, B43) and reverse transcription reaction (Thermo, K1622). Amplification and relative quantification of cDNA was carried out with SYBR^®^ Premix Ex Taq™ (Tli RNaseH Plus) (TaKaRa, RR420A) according to the manufacturer's protocol. Relative quantitative PCRs for miRNAs were performed with SYBR PrimeScript miRNA RT-PCR Kit (TaKaRa, RR716) as described before. Fold changes were calculated using the 2^−△△Ct^ method with normalization to endogenous control. Primers used were as follows: 5′-CTCGCTTCGGCAGCACA-3′ (U6-F), 5′-AACGCTTCACGAATTTGCGT-3′ (U6-R). miR-93 qPCR primers were purchased from RiboBio (Guangzhou, China). 5′-CCAGGCAGACATTGAGAACA-3′ (ULK1-F), 5′-GTTGGCAGCAGGTAGTCAGG-3′ (ULK1-R), 5′-CCACCCAGAAGACTGTGGAT-3′ (Gapdh-F), 5′-CACATTGGGGGTAGGAACAC-3′ (Gapdh-R). Independent experiments were performed in triplicate.

### 2.7. Western Blotting

Whole cell lysates used for immunoblotting were prepared in lysis buffer containing a phosphatase inhibitor (Roche, 4693116001) on ice. Lysates were mixed with SDS loading buffer and boiled for 10 min. Protein samples were separated on SDS-PAGE gels and then transferred to PVDF membrane. Membranes were blocked by 5% nonfat milk (dissolved in PBST) for 1 h at room temperature. Corresponding primary antibody was used for overnight incubation at 4°C, followed by HRP-labeled secondary antibodies' incubation at room temperature for 2 hrs. For loading controls, membranes were probed with the antibody against *β*-actin or Gapdh. Densitometric ratios were quantified by ImageJ software.

### 2.8. Immunofluorescence Microscopy

Cells were grown to 60% confluence on a coverslip. After treatment, the cells were washed in PBS and fixed with 4% paraformaldehyde for 20 mins. After washed twice in PBS, the cells were permeabilized with 0.1% Triton X-100 (in PBS). Coverslips were blocked in 1% albumin bovine V (in PBS) for 30 mins at room temperature and then were incubated with primary antibody diluted in PBS (0.01% Triton X-100) for 1 h at room temperature. After washed with PBS for 4 × 5 mins, secondary antibodies were applied. Cell images were captured with a TCS SPF5 II Leica confocal microscope and software (LAS-AF-Lite_2.2.0_4758, Germany).

### 2.9. CCK-8 Assay

Cell viability was measured using the CCK-8 kit. Briefly, 7 × 10^6^ cells in the logarithmic growth phase were seeded into a 24-well plate and incubated in 500 *μ*L complete medium overnight. MEFs and HeLa cells were transfected with scramble NC (NC), miR-93 mimics (93), NC inhibitor (IN-NC), or miR-93 inhibitor (IN-93) individually for 12 h, followed by the treatment of hypoxia for another 24 h. Moreover, MEFs were transfected with scramble NC (NC), miR-93 mimics (93), NC inhibitor (IN-NC), or miR-93 inhibitor (IN-93) individually for 24 h, followed by subjecting to starvation for another 1 h. The cell viability was detected according to the manufacturer's instructions. The optical density (OD) at 450 nm was measured using a microplate spectrophotometer (Biotek, Epoch). Cell viability was presented using the normal group as control.

### 2.10. Flow Cytometry

Cell apoptosis was also assessed by flow cytometry after Annexin V-FITC/PI staining as manufacturer's instructions. MEFs were transfected with scramble NC (NC), miR-93 mimics (93), NC inhibitor (IN-NC), or miR-93 inhibitor (IN-93) individually for 12 h, followed by the treatment of hypoxia for another 24 h. The normal group was set as control. Analyses were carried out with a COULTER EPICS XL-MCL™ flow cytometer (Beckman Coulter) equipped with Expo32 ADC analysis software. The cellular apoptosis rate was mapped by GraphPad Prism 5.

### 2.11. TUNEL Assay

Briefly, HeLa cells were transfected with scramble NC (NC), miR-93 mimics (93), NC inhibitor (IN-NC), or miR-93 inhibitor (IN-93) individually for 12 h, followed by the treatment of hypoxia for another 24 h. Analysis of apoptotic cells was performed using the TUNEL staining kit following the manufacturer's instruction, using normal group as control. Cell images were captured with an EVOS FL Auto Cell Imaging System (Thermo Fisher Scientific). The nuclei were counterstained with 4′,6-diamidino-2-phenylindole (DAPI) (blue). TUNEL-positive cells had a pyknotic nucleus with dark green fluorescent staining, indicative of apoptosis.

## 3. Results

### 3.1. miR-93 Targets ULK1 and Regulates Its Expression

**Figure 1:**
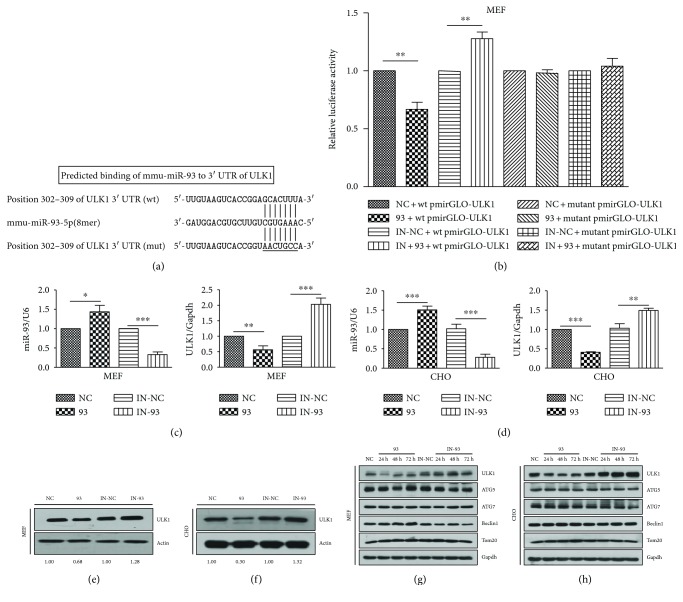
miR-93 targets ULK1 and regulates its expression. (a) Predicted binding sites of mmu-miR-93 to the 3′ UTR of ULK1. The short vertical lines indicate complementary paired bases of miR-93 and ULK1. The 302–309 nucleotides of ULK1 3′ UTR present the miRNA regulatory element (MRE). The underlined bases were mutant MRE. (b) Luciferase reporter assay by the interaction between miR-93 and the predicted MRE in MEFs. Each luciferase construct was cotransfected with scramble NC (NC), scramble IN-NC (IN-NC), miR-93 mimics (93), or miR-93 inhibitor (IN-93). At approximately 24 h after transfection, the luciferase activity was detected. The firefly luciferase activity was normalized to *Renilla*. Data were shown as the mean ± S.D. from three independent experiments. ^∗∗^*P* < 0.01. (c) miR-93 represses the endogenous ULK1 expression in MEFs. NC, 93, IN-NC, and IN-93 were transfected into MEFs. At 24 h after transfection, qPCR was performed to detect the expression of miR-93 and ULK1. Data were from three independent experiments. ^∗^*P* < 0.05; ^∗∗^*P* < 0.01; ^∗∗∗^*P* < 0.001. (d) miR-93 represses the endogenous ULK1 expression in CHO cells. NC, 93, IN-NC, and IN-93 were transfected into CHO cells. At 24 h after transfection, qPCR was performed to detect the expression of miR-93 and ULK1. Data were from three independent experiments. ^∗∗^*P* < 0.01; ^∗∗∗^*P* < 0.001. (e) Cell lysates in (c) were prepared and subjected to Western blot analysis by using anti-ULK1 and anti-*β*-actin antibody. The densitometric ratios of ULK1/actin from the samples were quantified by using ImageJ. Data were from three independent experiments. Representative data are shown. (f) Cell lysates in (d) were prepared and subjected to Western blot analysis by using anti-ULK1 and anti-*β*-actin antibody. The densitometric ratios of ULK1/actin from the samples were quantified by using ImageJ. Data were from three independent experiments. Representative data are shown. (g, h) MEFs and CHO cells were transfected with miR-93 (93) and miR-93 inhibitor (IN-93) for 24 h, 48 h, and 72 h, individually, setting group NC as a control. Cell lysates were prepared and subjected to Western blot analysis by using anti-ULK1, anti-ATG5, anti-ATG7, anti-Beclin1, anti-Tom20, and anti-Gapdh. Data were from three independent experiments. Representative data are shown.

ULK1 is the initiation factor of autophagy, which belongs to the family of serine/threonine kinases and is essential for the induction of autophagy [44, 45]. However, ULK1-modulating microRNAs are not well understood. In order to determine the miRNA that targets ULK1, we used DIANALAB to perform bioinformatics search. Interestingly, the software showed that ULK1 is the target of miR-93. Prediction of the interaction between miR-93 and 3′ UTR of ULK1 was shown in Figure 1(a). ULK1 contains a miR-93 binding site in the 3′ UTR (302–309 nucleotides). We then used the dual luciferase carrier pmirGLO to verify whether the miR-93 can identify and bind the 3′ UTR of ULK1. The 3′ UTR of ULK1 was cloned and inserted into the downstream of the firefly luciferase of plasmid pmirGLO. 100 nM scramble NC (NC), miR-93 mimics (93), NC inhibitor (IN-NC), or miR-93 inhibitor (IN-93) and a reporter gene were cotransfected into MEFs, respectively. As shown in Figure 1(b), the expression of miR-93 inhibited the firefly luciferase enzyme activity. However, the IN-93 could restore the activity of luciferase, suggesting the strong inhibitory effect of endogenous miR-93. Real-time quantitative PCR (qPCR) was performed by using U6 and Gapdh as the benchmark to detect the expression of miR-93 and ULK1 in either MEFs or CHO cells. The results showed that overexpression of exogenous miR-93 mimics or miR-93 inhibitor group was successful. ULK1 and miR-93 were individually inhibited by 93 and IN-93 (Figures 1(c) and 1(d)). The NC, 93, IN-NC, and IN-93 were also transfected into the cells for 24 h to detect the protein expression of ULK1 to confirm the inhibitory effect of miR-93 by Western blotting. miR-93, respectively, decreased the expression of ULK1 by 32% (in MEFs) and 70% (in CHO cells). Nevertheless, miR-93 inhibitor increased the expression of ULK1 by 28% (in MEFs) and 32% (in CHO cells) (Figures 1(e) and 1(f)). In order to exclude the off-target effects of miR-93 and to monitor a time course of the inhibitory effect of miR-93 to ULK1, some other autophagy-related genes were also examined by Western blotting. The expression of ATG5, ATG7, Beclin1, or Tom20 did not change when exogenously overexpressed with miR-93 and did not increase while exogenously overexpressed with miR-93 inhibitor (IN-93) (Figures 1(g) and 1(h)).

### 3.2. miR-93 Suppresses Hypoxia-Induced Autophagy

**Figure 2:**
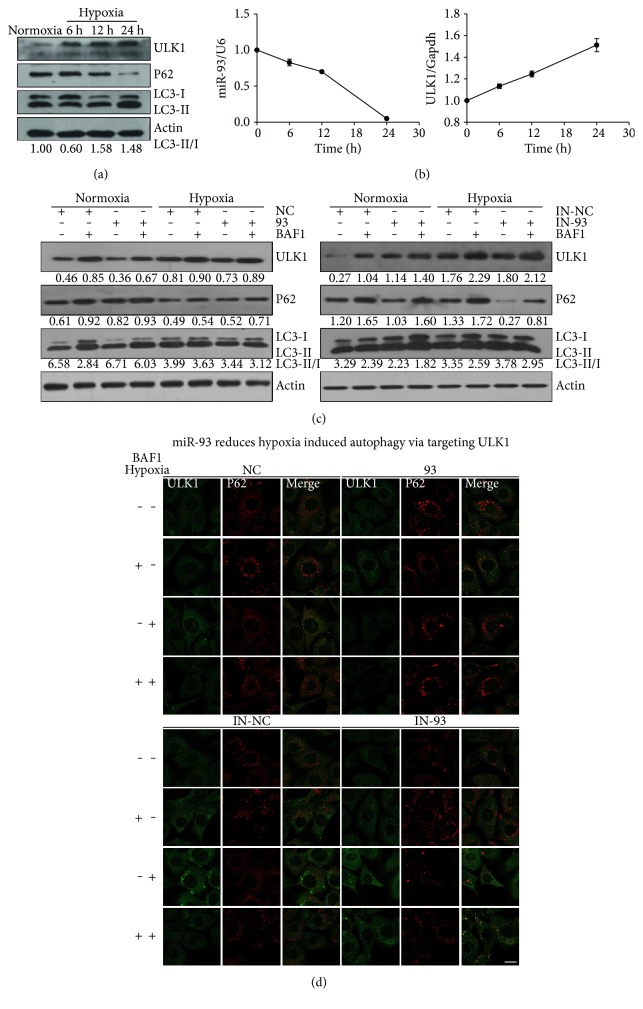
miR-93 inhibits hypoxia-induced autophagy. (a) The expression of ULK1 is induced by hypoxia. MEFs are subjected to hypoxia conditions for 0 h, 6 h, 12 h, and 24 h. Endogenous ULK1, P62, LC3, and *β*-actin were blotted by corresponding antibodies. Densitometric ratios of LC3-II/I from the samples were quantified by using ImageJ. (b) Expression levels of endogenous miR-93 and ULK1 in MEFs under hypoxia condition for 0 h, 6 h, 12 h, or 24 h by qPCR. (c) MEFs were transfected with scramble NC (NC), miR-93 mimics (93), scramble IN-NC (IN-NC), or miR-93 inhibitor (IN-93) for 12 h; afterward, MEFs were sealed in a hypoxic or normoxic condition for another 24 h with or without Bafilomycin A1 (BAF1). Samples were blotted with anti-ULK1, anti-P62, anti-LC3, and anti-*β*-actin antibodies. (d) MEFs were treated as the same as (c). Cells were fixed and immunostained with anti-ULK1 (green) and anti-P62 (red) antibodies. Scale bar, 20 *μ*m.

In order to find out whether miR-93 regulates the expression of ULK1 under the condition of hypoxia, cells were divided into four groups according to the time of hypoxia. Similarly as before, the protein levels of ULK1, P62, and LC3 transition confirmed that hypoxia could induce autophagy (Figure 2(a)). In hypoxia groups, the expression levels of ULK1 and LC3-I to LC3-II transition were increased but the expression of P62 was reduced (Figure 2(a)). The transcriptional and translational levels of ULK1 were higher than those in the normal group (Figures 2(a) and 2(b)). In contrast, the expression of miR-93 decreased during hypoxia (Figure 2(b)). Based on the above findings, we hypothesized that miR-93 might play an important role in hypoxia-induced autophagy. We then transfected scramble NC (NC), miR-93 mimics (93), NC inhibitor (IN-NC), or miR-93 inhibitor (IN-93) to examine their roles in hypoxia-induced autophagy. We found that miR-93 caused the downregulation of ULK1 as well as upregulation of P62, and decreased the ratio of LC3-II to LC3-I under hypoxic condition (Figure 2(c)). In contrast, this effect could be reversed by the endogenously overexpressed IN-93 during hypoxia. The expression of ULK1 in miR-93 group was lower than that in NC group. Compared to IN-NC group, IN-93 group promoted the expressional level of ULK1 (Figure 2(c)). Lysosomal inhibitor Bafilomycin A1 (BAF1) prevented the transition of LC3 and P62 protein degradation under hypoxic condition, indicating that hypoxia promotes the improvement of autophagic flux. The densitometric ratios from the samples were quantified by using ImageJ (Figure 2(c)). The results were further examined by immunofluorescence. The samples were stained with anti-ULK1 (green) and anti-P62 (red) antibodies. miR-93 inhibited the expression of ULK1 as well as its puncta formation during hypoxia (Figure 2(d)). On the contrary, miR-93 increased the P62 puncta under the same condition. Conversely, this effect could be reversed by IN-93, an inhibitor of endogenous miR-93. Upon inhibition of endogenous miR-93, ULK1 puncta increased accompanied by decreased expressional level of P62 (Figure 2(d)).

### 3.3. The Knockdown of ULK1 by siRNA Suppressed Hypoxia-Induced Autophagy

**Figure 3:**
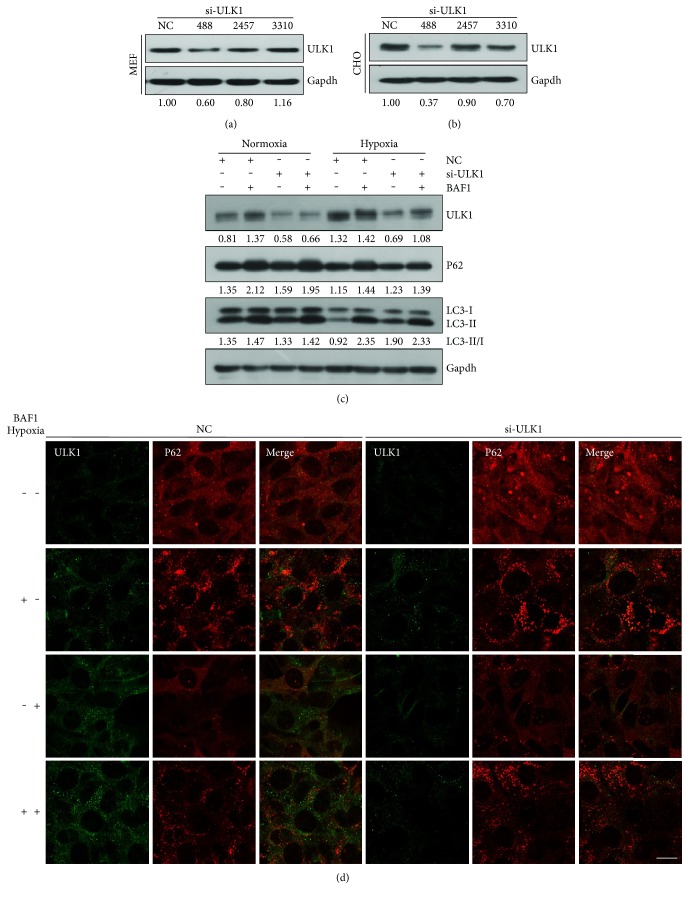
The knockdown of ULK1 by siRNA suppressed hypoxia-induced autophagy. (a, b) MEFs and CHO cells were transfected with three pairs of siRNAs to ULK for 24 h, respectively, si-ULK1 (488), si-ULK1 (2457), and si-ULK1 (3310), setting NC as a control. Cell lysates were prepared and subjected to Western blot analysis by using anti-ULK1 and anti-Gapdh. The densitometric ratios from the samples were quantified by using ImageJ. Data were from three independent experiments. Representative data are shown. (c) Si-ULK1 (488) was transfected into MEFs for 12 h, followed by hypoxic treatment or normoxic treatment for another 24 h. Cell lysates were analyzed by Western blot using anti-ULK1, anti-P62, anti-LC3, and anti-Gapdh. Densitometric ratios of the samples were quantified by using ImageJ. (d) MEFs were treated as the same as (c). Cells were fixed and immunostained by anti-ULK1 (green) and anti-P62 (red) antibodies. Scale bar, 20 *μ*m.

From the above results, we knew that hypoxia could trigger the decrease of miR-93 and the increase of ULK1 expression and consequently induce autophagy. Therefore, the modulatory effect of miR-93 on ULK1 might become less relevant since the weakened expression of miR-93 under hypoxic condition. To determine the relevance of miR-93 in the regulation of ULK1, siRNA of ULK1 was used to reveal that if knocking down of ULK1 had the same effect similar to miR-93 in hypoxia-induced autophagy. It is clear that siRNA, a class of double-stranded RNA molecules, interferes with the expression of specific genes by degrading mRNA after transcription (via complementary nucleotide sequences), resulting in no translation [46, 47]. Four pairs of siRNAs (including negative control) were designed, synthesized, and transfected into MEFs and CHO cells. Fortunately, we found the most effective siRNA interference site (488) attributing to its interference efficiency in MEF cells (~40%) and CHO cells (~60%) (Figures 3(a) and 3(b)). We then transfected si-ULK1 (488) and negative control RNA (NC) to examine their roles in hypoxia-induced autophagy. We found that si-ULK1 caused the downregulation of ULK1 as well as upregulation of P62 and decreased the ratio of LC3-II/LC3-I under hypoxic condition. The expression of ULK1 in si-ULK1 group was lower than that in NC group. BAF1 prevented the transition of LC3 and P62 protein degradation under hypoxic condition. The densitometric ratios from the samples were quantified by using ImageJ (Figure 3(c)). The results were further examined by immunofluorescence. The samples were stained with anti-ULK1 (green) and anti-P62 (red) antibodies. si-ULK1 inhibited the expression of ULK1 as well as its puncta formation during hypoxia. On the contrary, si-ULK1 increased the P62 puncta under the same condition (Figure 3(d)).

### 3.4. Re-Expression of ULK1 without miR-93 Binding Elements Restores Autophagy Inhibited by miR-93

**Figure 4:**
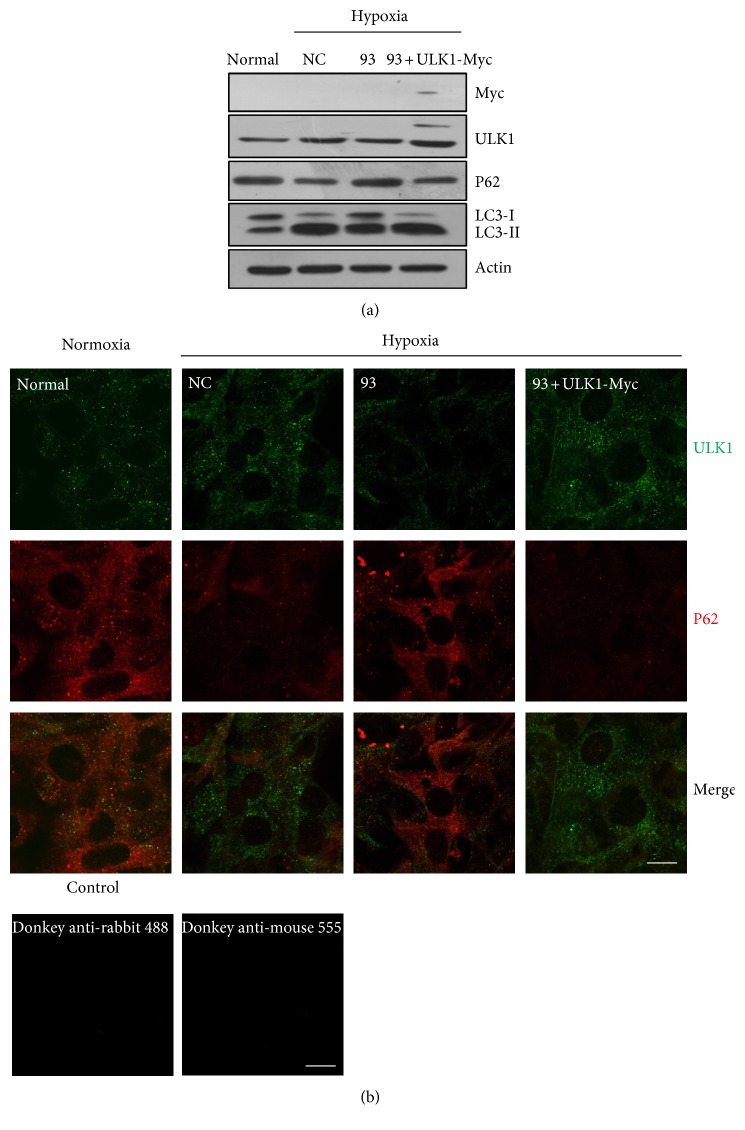
Re-expression of ULK1 without miR-93 response elements restores autophagy inhibited by miR-93. (a) MEFs were transfected with scramble NC (NC) and miR-93 mimics (93) or cotransfected miR-93 mimics (93) with the indicated constructs (ULK1-Myc) for 24 h by using the normal group as a control. Protein samples were collected and the expression of ULK1, Myc, LC3, and P62 was analyzed through Western blot. Actin was used as a loading control. (b) MEFs were fixed and immunostained by donkey anti-rabbit 488 or donkey anti-mouse 555. Scale bar, 20 *μ*m. (c) MEFs were treated the same as (a). Cells were fixed and immunostained by anti-ULK1 (green) and anti-P62 (red) antibodies. Scale bar, 20 *μ*m.

In order to verify that the inhibition of ULK1 is due to miR-93, we evaluated the expression of ULK1-Myc (CDS) which lacked the miR-93 recognition element so that it can resist miR-93-mediated inhibition. ULK1-Myc (CDS) transfection effectively induced the P62 degradation and LC3-I to II transition even in the presence of miR-93 under hypoxia (Figure 4(a)). In order to distinguish the immunoreactivity-derived signal from the background staining, MEFs were fixed, permeabilized, and only stained with the second antibody donkey anti-rabbit 488 or donkey anti-mouse 555. As seen from Figure 4(b), there was no fluorescence signal of MEFs. Consistent with Figure 4(a), immunofluorescence results revealed that ULK1-Myc could effectively induce P62 degradation which was inhibited by exogenous overexpression of miR-93 under hypoxia as confirmed by WB (Figure 4(b)).

### 3.5. The Effects of miR-93 on Cell Viability and Apoptosis in Noncancer Cell Lines and Cancer Cells

**Figure 5:**
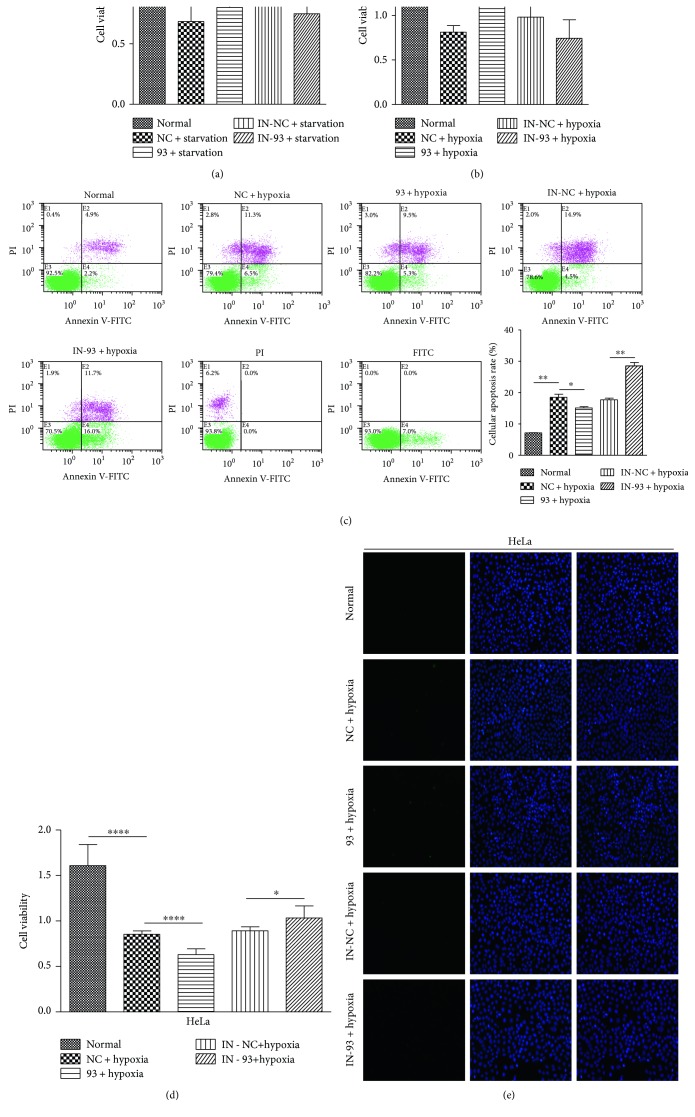
The effects of miR-93 on cell viability and apoptosis in noncancer cell lines and cancer cells. (a, b) CCK8 proliferation assays were performed on MEFs transfected with indicated RNA for 12 h. MEFs were exposed to starvation or hypoxic conditions for another 24 h. The graph represents the cell proliferation of three replicates with standard deviation represented by error bars. ^∗∗^*P* < 0.01; ^∗∗∗^*P* < 0.001. (c) MEFs were transfected with the indicated microRNAs or left untransfected for 12 h; the cells were exposed to hypoxic condition for another 24 h. The cell apoptosis rate was detected by flow cytometry. Cellular apoptosis rate was calculated by GraphPad Prism 5. (d) HeLa cells were transfected with scramble NC (NC), miR-93 mimics (93), scramble IN-NC (IN-NC), or miR-93 inhibitor (IN-93) for 12 h, followed by hypoxic treatment for another 24 h. Normal group was set as control. The CCK8 proliferation assays were detected. ^∗^*P* < 0.05; ^∗∗∗∗^*P* < 0.0001. (e) HeLa cells were treated as (d), then subjected to TUNEL staining. DAPI (blue), TUNEL-positive cells (green). Magnification, 20x.

Some genes involved in cell death are associated with the autophagy pathway. Excessive autophagy leads to cell death due to excessive digestion of essential intracellular proteins and organelles, which triggers the death signal [48–50]. Considering that miR-93 is involved in hypoxia by targeting key autophagy proteins, we assessed whether miR-93 is involved in cell viability in nutrient-depleted condition or during hypoxia. We used the cell counting kit to analyze and evaluate the cell viability during either condition. The cell viability of MEFs decreased to about 65%, in these conditions (Figures 5(a) and 5(b)). Upon starvation, the cell viability of MEFs did not change notably after the exogenous overexpression of miR-93 or miR-93 inhibitor, compared to NC or IN-NC group. Although the cell viability of MEFs between NC + starvation group and IN-NC + starvation group seemed to be different, there was no significant difference between the two groups (*P* = 0.09) (Figure 5(a)). Unlike starvation condition, in hypoxic condition, the miR-93 group recovered the cell viability from 65% to 136% (±6%) compared with NC group, while IN-93 further dampened the cell viability of MEFs to about 74% (±21%) during hypoxia, (Figure 5(b)). Although the cell viability of MEFs between NC + hypoxia group and IN-NC + hypoxia group seemed to vary, there was no significant difference between the two groups (*P* = 0.21) (Figure 5(b)). We subsequently examined whether miR-93 protected the cells from hypoxic stress by weakening cell apoptosis. Flow cytometry analysis revealed that miR-93 decreased hypoxia-induced cell apoptosis of MEFs, which was reversed by miR-93 inhibitor (IN-93) (Figure 5(c)). Given that autophagy and hypoxia are implicated to play an important role in cancer, we further investigated miR-93-based regulation of ULK1, and hypoxia-mediated modulation of miR-93 in human cancer cells. HeLa cells were used to take cell viability test and TUNEL assay. As shown in Figure 5(d), the cell viability of HeLa was dramatically reduced under hypoxic condition, compared with normal group. Interestingly, the exogenous overexpressed miR-93 (93) further accelerated the decrease of cell viability under the stress of hypoxia. By contrast, miR-93 inhibitor (IN-93) could reverse the cell viability by reducing the expression of endogenous miR-93. In view of the interesting experimental phenomena mentioned above, we did the TUNEL assay afterwards. Nevertheless, the overall apoptosis differences between groups were not significant (Figure 5(e)).

## 4. Discussion

Here, we found that the expression of ULK1 was upregulated but miR-93 level was declined under hypoxic condition. Their expression patterns showed a negative correlation (Figure 2). By using LAB DIANA, we uncovered a direct relationship of miR-93 and ULK1. The 3′ UTR of ULK1 had miR-93 pairing sites. The luciferase activity assay, quantitative RT-PCR, and Western blotting results showed that miR-93, when overexpressed, regulated ULK1 at the transcriptional and posttranscriptional levels. The inhibitory effect of miR-93 on ULK1 was also observed under hypoxic condition (Figure 2). Immunofluorescence showed that the expression of ULK1 was declined and the P62 level was increased by miR-93 (Figure 2). To determine the relevance of miR-93 in the regulation of ULK1, siRNA of ULK1 was used to reveal that knockdown of ULK1 had the same effect as compared to miR-93 in hypoxia-induced autophagy. Data showed that hypoxia induced autophagy was indeed suppressed after interference with ULK1 (Figure 3). This is consistent with the results from miR-93. In order to verify that the inhibition of ULK1 was caused by miR-93, we assessed whether the ULK1 plasmid lacking the miR-93 recognition element was able to resist miR-93-mediated inhibition. Immunofluorescence and Western blotting indicate that ULK1 plasmid that lacks miR-93 recognition element can rescue the autophagy inhibited by miR-93 (Figure 4).

Substantial progress helps us to understand the molecular mechanism of autophagy but there are still a lot of unknowns needed to be further clarified. Recent evidence that most autophagy genes can be regulated by miRNAs increases a more complicated cellular layer control of autophagy. Autophagy and microRNAs seem to have mutual communications with each other. On one hand, microRNAs regulate autophagy by directly targeting the autophagy genes including Beclin1 [51, 52], ATG3 [53], or ATG5 [54] as well as mitophagy genes, FUNDC1 and NIX [29]. On the other hand, autophagy activity modulates miRNA-mediated gene silencing and degrades the core miRISC component [55].

We found the negative correlation of miR-93 and ULK1. While ULK1 is induced and miR-93 is decreased under hypoxia. Interestingly, Hazarika et al. reported that hypoxia induced an increased expression of miR-93 [56], but Ke and colleagues observed a decreased level of miR-93 [41], which is consistent with our observations. We thought that the discrepancy may be due to the use of different cell types.

The cellular level of ULK1 is coordinately controlled by a variety of mechanisms. During early stages of autophagy, the E3 ligase complex AMBRA1–TRAF6 mediates K63-linked polyubiquitination of ULK1 to maintain its stability and kinase activity [57]. On the contrary, Cullin/KLHL20 catalyzes K48-linked polyubiquitination of ULK1 for proteasome degradation during prolonged nutrient starvation, thus providing negative feedback loop of the autophagy control [17]. Recently, Nazio et al. provide evidence that levels and activity of ULK1 are temporally controlled by NEDD4L-mediated degradation and mTOR-dependent de novo protein synthesis to modulate the duration of the autophagy response during prolonged starvation [19]. Similarly, the ULK1 expression is induced under hypoxia treatment. Our previous results demonstrate that the level of ULK1 is regulated by hypoxia from tens of minutes to 24 hours [15, 16]. But the expression of ULK1 declines if the hypoxic time is longer than 36 hours, indicating that the ULK1 expression is controlled by multiple cellular factors.

In this study, we have discovered that ULK1 is a new target gene of miR-93. Unlike the effect of miR-93 in nutrient-deprivation condition, our results show that miR-93 was able to recover the cell viability and lower the rate of apoptosis compared to the NC group under hypoxic condition in noncancerous MEFs, indicating that miR-93 might contribute some beneficial effects to this kind of cells under hypoxia. However, the effect of miR-93 in cancerous cell line HeLa is quite different. The cell viability of HeLa was dramatically reduced under hypoxic condition, compared with normal group. But, the exogenous overexpressed miR-93 (93) further accelerated the decrease of cell viability under the stress of hypoxia. By contrast, miR-93 inhibitor (IN-93) could revert the cell viability by reducing the expression of endogenous miR-93. The data suggests that autophagy might be more important for sustaining the viability of the cancerous cell line than noncancer cell lines during stressful hypoxia condition. Nevertheless, the overall apoptosis differences between groups were not significant (Figure 5). The different cell viability results of miR-93 in cancerous and noncancerous cell lines under hypoxia condition demonstrated a quite complex regulation of microRNA, autophagy, and cells that undergo hypoxic stress. This may be because of that the metabolism and oxygen consumption rate varies in cancerous and noncancerous cell lines. The detailed molecular mechanism needs to be further investigated.

We also observed that hypoxia substantially increases autophagy flux by upregulating the levels of ULK1 and suppresses the expression of miR-93 (Figure 2). This study provides a novel insight into how the oscillation level of ULK1 is maintained by miR-93, and miR-93-modulated key autophagic machinery involved in hypoxic microenvironment.

## Supplementary Material

Supplementary Figure S1. Transfection efficiency detection of MEFs and CHO cells. A. GFP vectors were transfected into MEFs and CHO cells for 24 h. Cell images were captured with a EVOS FL Auto Cell Imaging System (Thermo Fisher Scientific). GFP-positive transfected cells (green). The nuclei were stained with DAPI (blue). B. Negative control FAM (FAM-siRNA NC) was transfected into MEFs and CHO cells for 24 h. Cell images were captured with a EVOS FL Auto Cell Imaging System (Thermo Fisher Scientific). FAM-siRNA NC (green), DAPI (blue).
