# Supplementary figures and images for: MicroRNA-93 Regulates Hypoxia-Induced Autophagy by Targeting ULK1
Source: Oxid Med Cell Longev. 2017 Oct 3;2017:2709053. doi: 10.1155/2017/2709053 (PMC5646326; doi:10.1155/2017/2709053)

Supplemental Fig1. Transfection efficiency detection of MEFs and CHO cells

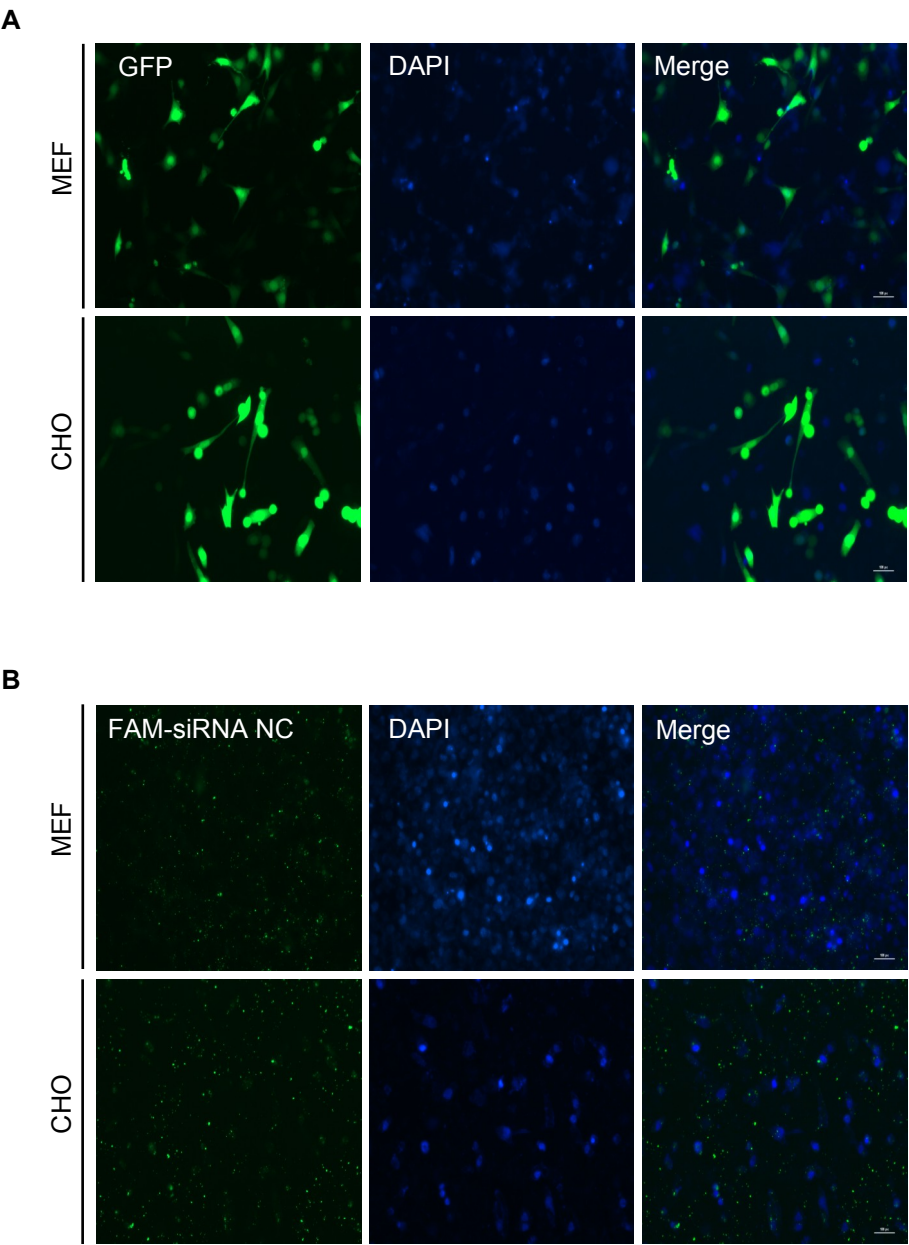

Supplement: Supplementary file 1 — Supplementary Figure S1. Transfection efficiency detection of MEFs and CHO cells. A. GFP vectors were transfected into MEFs and CHO cells for 24 h. Cell images were captured with a EVOS FL Auto Cell Imaging System (Thermo Fisher Scientific). GFP-positive transfected cells (green). The nuclei were stained with DAPI (blue). B. Negative control FAM (FAM-siRNA NC) was transfected into MEFs and CHO cells for 24 h. Cell images were captured with a EVOS FL Auto Cell Imaging System (Thermo Fisher Scientific). FAM-siRNA NC (green), DAPI (blue). [file 2709053.f1.pdf]
